# Effects of high summer temperatures on mortality in 50 Spanish cities

**DOI:** 10.1186/1476-069X-13-48

**Published:** 2014-06-09

**Authors:** Aurelio Tobías, Ben Armstrong, Antonio Gasparrini, Julio Diaz

**Affiliations:** 1 Institute of Environmental Assessment and Water Research (IDAEA), Spanish Council for Scientific Research (CSIC), C/Jordi Girona 18-26, Barcelona 08031, Spain; 2 Department of Social and Environmental Health Research, London School of Hygiene and Tropical Medicine (LSHTM), 15-17 Tavistock Place, London WC1H 9SH, UK; 3 Department of Medical Statistics, London School of Hygiene and Tropical Medicine (LSHTM), Keppel Street, London WC1E 7HT, UK; 4 National School of Public Health (ENS), Instituto de Salud Carlos III (ISCIII), Madrid, Avda. Monforte de Lemos 5, Madrid 28029, Spain

**Keywords:** Temperature, Heat, Mortality, Spain, Time-series, Heterogeneity, Adaptation

## Abstract

**Background:**

Periods of high temperature have been widely found to be associated with excess mortality but with variable relationships in different cities. How these specifics depend on climatic and other characteristics of cities is not well understood. We assess summer temperature-mortality relationships using data from 50 provincial capitals in Spain, during the period 1990–2004.

**Methods:**

Poisson time series regression analyses were applied to daily temperature and mortality data, adjusting for potential confounding seasonal factors. Associations of heat with mortality were summarised for each city as the risk increments at the 99th compared to the 90th percentiles of the whole-year temperature distributions, as predicted from spline curves.

**Results:**

Risk increments averaged 14.6% between both centiles, or 3.3% per 1 Celsius degree. Although risk increments varied substantially between cities, the range of temperature from the 90th to 99th centile was the only characteristic independently significantly associated with them. The heat increment did not depend on other city climatic, socio-demographic and geographic determinants.

**Conclusions:**

Cities in Spain are partially adapted to high mean summer temperatures but not to high variation in summer temperatures.

## Background

It has been shown that daily mortality increases when air temperatures are unusually high for the area [1-5] and the association between mortality and temperature has also been identified as non-linear, showing a J- or V-shape relationship [6-8]. However, important details remain unclear, in particular, how temperature-mortality associations vary across populations depending on differing climatic, demographic and socioeconomic profiles [9]. The variation between regions [2,5,6,10,11] suggests adaptation to some degree of populations to local climate, but the specifics of how much and why associations differ by cities is not known. Several studies have found that minimum mortality temperature increases with a city’s mean temperature [8,10,11] but those that have quantified this association or explored climatic variables as determinants of other aspects of the association are fewer [5,6,11]. Heat thresholds, population density, percentage of people aged 65 or more, and economic deprivation have been independently associated with an increase in the heat slope [9].

Given impending climate change and the traumatic experience of the 2003 heat wave in Europe, most attention is focused on heat. In Spain overall, only the effect of *administratively* defined heat waves has been addressed nation-wide [12], concluding that there is a need for an understanding of the temperature-mortality association beyond pre-defined *waves*, as well as a general scientific and public health need to elucidate determinants of variation between and how it varies between Spanish cities. The availability in Spain of data for a large number of cities with similar health and data collection systems give these data several advantages addressing these issues.

The objective of our study was to assess the relationship between high temperatures and total daily mortality in provincial capital of Spain. We assessed climatic, socio-demographic and regional determinants that may explain variation in city-specific associations.

## Methods

### Data

Our study includes data for all provincial capital cities in Spain for the period 1990–2004. Spain is divided in 17 Autonomous Regions, each composed from one up to nine provinces, and each province has a capital city. Spanish territory also includes the Balearic Islands in the Mediterranean, the Canary Islands in the Atlantic Ocean, and two Autonomous cities in Northern Africa (Ceuta y Melilla). Data on total daily all-cause mortality during summer (1 June – 31 September), excluding accidents (International Classification of Diseases-9th revision/ICD-9: 1–799), for the 52 provincial capital were provide by the Spain National Institute of Statistics. Daily mean, minimum and maximum temperatures (in °C) were collected from the Spain National Meteorology Agency. In two provincial capital cities, Palencia and Ceuta, daily temperatures were not available.

### Statistical analysis

We estimated a city-specific relationship between daily mortality in summer and maximum temperature using an over dispersed Poisson Generalised Estimating Equation model (GEE). Maximum temperature was chosen as the index providing best fit in previous single city-studies conducted in Spain cities [13-15]. A lagged effect for up to two days, after that heat effects declined has also been shown [12]. Thus, we considered the average of the maximum temperature for the same day and up to two days previous. Applying the methodology used in previously conducted European multi-city studies [2,5,16], we modelled the marginal relationship between total daily mortality and maximum temperature over average lag 0–2 by means of 4 degrees of freedom (df) natural cubic splines (NCS), also specifying a first-order autoregressive correlation structure within summer in the Poisson GEE model. Long-term trends were modelled using NCS of the variable time allowing 1 df for every 5-years of data. To allow for within summer seasonal variation not explained by extreme heat days, we fit a 4 df NCS of day of year constrained to be the same over all years. The above model was fitted separately to each capital city. To summarise the effect of relative temperature change at high temperatures, we calculated from the fitted spline the change in mortality risk comparing the 99th to 90th percentile of the capital city’s year-round maximum temperature distribution. This summary of the heat effect derived from the overall non-linear exposure-response curve has been used previously, so provides a benchmark that can be compared [9]. Finally, we transformed this to an increment per 1°C by dividing the overall above risk increment by the difference between the 99th and 90th percentiles to allow comparisons with other studies reporting slopes of heat-mortality associations as increment per 1°C [2,5,6,8,10]. To explore sensitivity of the above results to model assumptions, in particular where others have been used [5,9], we considered alternative lag structures from of the same day and the average up to a previous week and again estimated increase in risk from 90th to 99th percentile.

In a second stage of the analysis, city-specific estimates of relative heat effects were combined to generate a national estimate using random effects meta-analysis [17]. To explore heterogeneity of effects across cities, random-effects meta-regression models [18] were fitted with variables at city level including geographic (altitude, latitude, longitude and surface), socio-demographic (total population, proportion of population older than 65 years and per capita income) and climatic characteristics (yearly and summer temperatures, and relative humidity). The analysis of heterogeneity was conducted for continental Spain, excluding the three non-continental cities; Las Palmas and Tenerife (in Canary Islands), and Melilla in Northern Africa), since their geographic and climatic characteristics are completely different from continental cities.

All analyses were done using Stata 12 (StataCorp LP, College Station, TX, 2011).

## Results

### City-specific mortality risk and combined analysis

**Figure 1:**
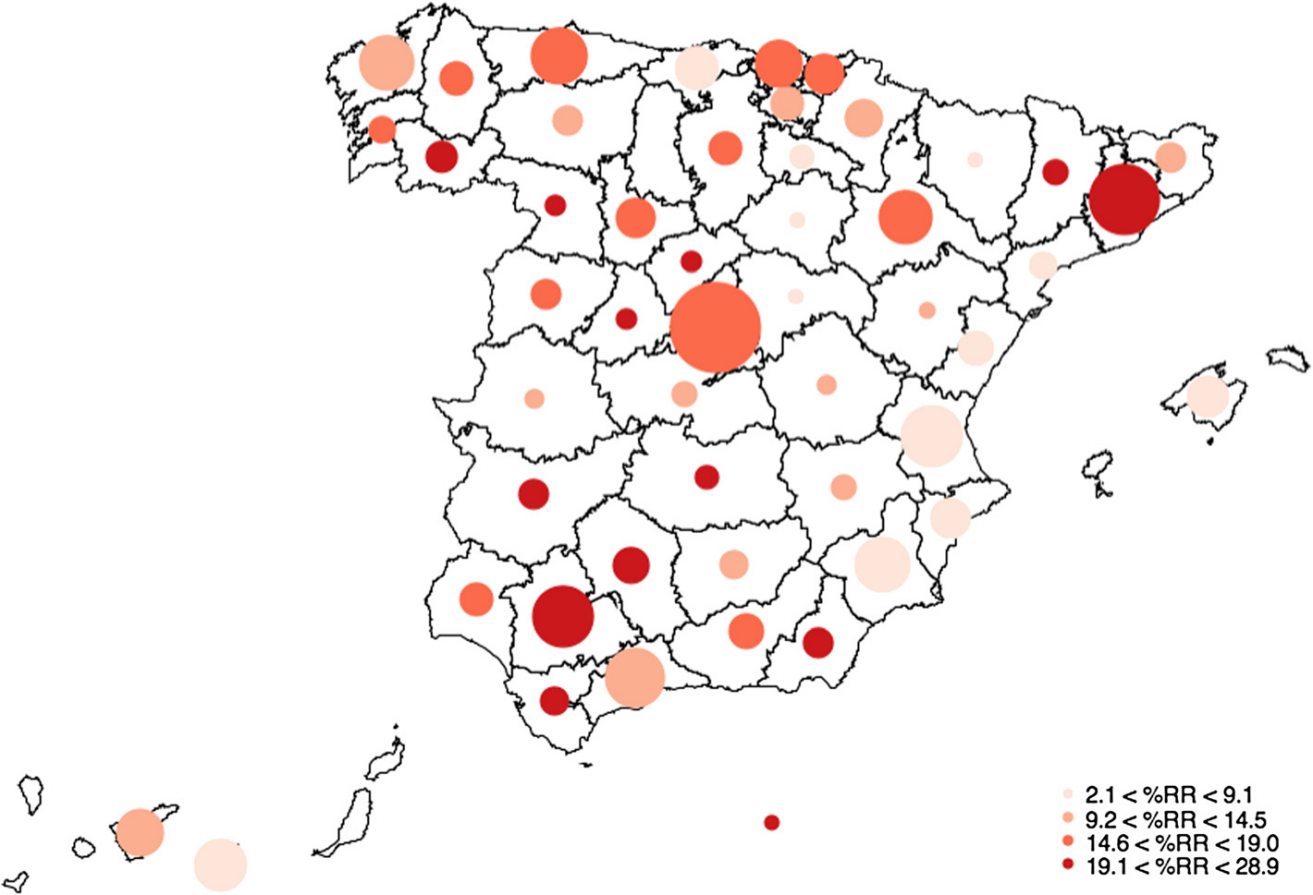
**Geographical distribution of the city-specific percentage relative risk (%RR) of mortality comparing the 99th to 90th percentile of maximum temperature.** The size corresponds to the inverse of the variance of the risk estimate (i.e., larger circles are more certain).

The city-specific exposure-response curves were V or J shaped for most cities showing clear evidence of increased mortality at the highest temperatures for most of the cities (Additional file 1). The difference between the 99th and 90th percentiles of daily maximum temperature was on average 4.3°C, ranging from 2.4°C in Alicante (Southeast Mediterranean) to 6.3°C in Lugo (Northwest). The overall average increase in mortality was 14.6% (95% confidence interval = 12.5% to 16.8%). Estimates between cities showed significant heterogeneity (I^2^ = 76.8%, p < 0.001). When the mortality risk was expressed per 1°C the average increase of risk was 3.3% (95% CI = [2.8%, 3.7%]) for a 1°C, also showing a large though somewhat smaller heterogeneity (I^2^ = 71.1%, p < 0.001). City-specific mortality risk estimates for 99th vs. 90th percentiles and for 1°C the average increase are reported in Additional file 2. Geographical distribution of city-specific mortality risks at high temperatures (99th vs. 90th percentiles) is shown in Figure 1. The geographical pattern suggested a trend from East to West, with higher risks at Southern and Western regions than the Mediterranean area. Although there were exceptions, like for the city of Barcelona that showed a much largest risk of 27% (95% CI = [23%, 32%]) than other Mediterranean cities.

In a sensitivity analysis, the risk of mortality raised from 9% (95% CI = [7.4% to 10.7%]) for the same day, to 12.5% (95% CI = [10.6% to 14.5%]) for the average lag 0–1, both lower than the 14.6% given above for average lag 0–2. For longer averages the risk increments declined from 14.6%. When in another sensitivity analysis we excluded the year 2003, the risk increment for average lag 0–2 was slightly lower, 11% (95% CI = [9.5% to 12.5%]).

### Analysis of heterogeneity

Effect modification by specific-city characteristics, estimated by random effects meta-regression, is shown in Table 1. To present results for these models for easier interpretation, we report the effect of temperature at the 25th and 75th percentiles of each city-characteristic (Additional file 3), as predicted for the meta-regression model, and testing for differences between both estimates. The difference between the 99th and 90th percentiles of maximum temperature, that is the range over which the heat effect was estimated, was the most significantly related with the heat effect (p = 0.011), with larger risks for larger temperature differences. The heat’s relative effect ranged from 14% (95% CI = [11.9%, 16%]) at the 25th percentile of 4°C (that is, for example, in a city like Malaga –South–) up to 16.4% (95% CI = [14.2%, 18.7%]) at the 75th percentile of 4.8°C (Bilbao –North–). Apart from this, only the longitude was statistically significant (p = 0.039), showing a change in the heat’s relative effect from 16.7% (95% CI = [14.1%, 16.4%]) at the 25th percentile of 5.1 West (Seville –South– or Salamanca –North–) down to 13.5% (95% CI = [11.2%, 15.9%]) at the 75th percentile of 1.2 West (Murcia –South– or Pamplona –North–). However, longitude and difference of maximum temperature percentiles were negatively correlated (Additional file 4, r = −0.4) indicating that difference between hot temperatures was larger in Western cities. When adjusting simultaneously for both variables in a multivariate random effects meta-regression, only the difference of maximum temperature percentiles remained marginally significant (p = 0.089).

**Table 1:** Heat-effect estimates at 25th and 75th percentiles of city-specific geographic, socio-demographic and climatic characteristics according to random effects meta-regression analysis

		**At p25 of characteristic**			**At p75 of characteristic**		
**City-characteristics (units)**	**p25**	**%RR**^**a**^	**(95% CI)**	**p75**	**%RR**^**a**^	**(95% CI)**	**p**^**b**^
Heat exposure							
p99th-p90th of maximum temperature (°C)	4.0	14.0	(12.0, 16.0)	4.8	16.4	(14.2, 18.7)	0.011
Geographic							
Latitude	38.6	15.7	(13.1, 18.4)	42.2	14.0	(11.3, 16.8)	0.318
Longitude	−5.4	16.7	(14.1, 19.4)	−1.2	13.5	(11.2, 15.9)	0.039
Socio-demographic							
Population (×100,000 hab.)^c^	0.7	14.2	(11.3, 17.2)	6.7	15.1	(13.0, 17.3)	0.480
% Pop. >65 years^c^	0.1	14.7	(12.0, 17.5)	0.2	15.1	(12.5, 17.8)	0.820
Per capita income (×1,000 €)^d^	8.6	15.9	(13.2, 18.7)	11.3	14.1	(11.6, 16.7)	0.273
Climate							
Whole year							
Mean temperature (°C)	13.1	14.9	(12.1, 17.8)	17.2	15.0	(12.4, 17.6)	0.967
Relative humidity (%)	61.3	15.7	(12.9, 18.6)	70.6	14.4	(11.9, 16.9)	0.414
Summer (Jun. To Sep.)							
Mean temperature (°C)	19.6	14.5	(11.7, 17.4)	24.6	15.3	(12.6, 18.1)	0.679
Relative humidity (%)	48.9	16.5	(13.6, 19.4)	66.5	13.9	(11.6, 16.3)	0.158

^a.^Percentage of relative risk of mortality comparing the p99 vs. p90 of max. temp.

^b.^p for comparison of change in mortality at p75 vs. p25.

^c.^Source: Demographic Information System, Municipal Register 1999, National Statistics Institute.

^d.^Source: Spanish Regional Accounts, Base 2000, National Statistics Institute.

## Discussion

Our results quantify the impact that high temperatures have on summer daily mortality in Spain. Similarly positive associations have also been found in other European [2-5,16,19,20] and US [1,6,11] multi-city studies. This study adds importantly to evidence on the sizes of heat effects worldwide, thus enabling explorations of how and why they differ. Although in Spain effects of high temperatures had been investigated in single cities [8,13-15] and in some specific regions [21,22], a national study of heat effects in all major Spanish cities has not previously been done. Only the effect of *administratively* defined heat waves was recently addressed [12] nation-wide, concluding that there was a need for an understanding of the temperature-mortality association beyond pre-defined *waves*. The correlation between the heat effects reported in this study and the effect of *administratively* defined heat waves is r = 0.4, rising up to r = 0.6 when only considering those cities when heat waves thresholds were not arbitrarily modified [12]. The health benefits of implementing heat-wave prevention plans are undeniable. It is also evident that these must be optimised through in-depth knowledge of the relationship between high summer temperature and health as well as the geographic, socio-economic and climatic characteristics that influence the city-specific relationship, as those reported in this study.

The overall average increase in mortality of 14.6% found in our study is much larger than the 3% previously reported in a multi-city study conducted in US also comparing the risk of relative changes between the 99th and 90th percentiles of maximum temperature [11]. Other multi-city studies conducted in Europe have reported risk of mortality by 1°C increase of maximum or mean apparent temperatures. A European study [2] showed a risk of 3.1% for a 1°C increase over a threshold of 29.4°C in the Mediterranean area (which included the cities of Athens, Rome, Barcelona, Valencia, Turin, Milan and Ljubliana) which agrees with the 3.3% for a 1°C increase between the 90th and 99th percentiles of maximum temperature found in our study. Furthermore, overall estimates for CIRCE study, combining data from ten Southern Mediterranean cities, was around 4% in all causes of mortality for 1°C increase above maximum apparent temperature threshold [5]. Similar estimates were also found in studies conducted in two Portuguese cities [19] also in ten government regions of England and Wales [3] reporting risks for a 1°C increase of mean apparent temperature of 2.1% during warm season (from April to September) over the year-round 93^rd^ percentile. Lastly, a larger effect of 5% for a 1°C increase over the 90th percentile of mean summer temperature was found in a study conducted in three regions of Sweden [20].

The way to address for the relationship between high temperature and mortality had varied between studies, from linear thresholds models that estimates a constant linear relationship below and above the minimum temperature mortality [6,7] to others with constant slopes above and below specific city thresholds for hot temperatures [3]. However, when comparing cities with different climates that may not reflect the true city-specific temperature-mortality relationship. We used a spline approach to allow for estimation of non-linear relationships without forcing constant slopes among cities [5,6,11]. Since some cities did not seem to have a clear minimum mortality temperature and city-specific thresholds for heat varied considerably among cities, we summarised the effect of relative temperature change in mortality risk comparing the 99th to 90th percentile of the capital city’s year-round maximum temperature distribution [11]. We investigated lagged effects form same day up to a previous week, founding the strongest association between for the same and up to two previous days. Most studies have also reported the same short lagged effects [1,3,5,11] suggesting a rapid physical response to the heat effect. Furthermore, we also did explore many other geographic (surface, altitude and coastal), socio-demographic (population density, annual household income, average expenditure by household, at risk of poverty rate, average capacity of hospitals, percentage of hospital occupation and hospital purchases and expenses per inhabitant) and climatic (whole year and summer minimum and maximum temperatures, standard deviation for whole year and summer temperatures, monthly hours of sunshine, rainfall and atmospheric pressure) variables. However, very similar results were obtained since most of these variables were highly correlated (r > 0.7). Thus, in terms of parsimony we only reported those variables comparable with previously published multi-city studies [5,9].

The geographical variability in the distribution of the city-specific estimates was mainly driven by the difference between the 99th and 90th percentiles of maximum temperature, which was associated with raising the risk of mortality due to heat by 2.4% for an interquartile range increases in this difference of 0.8°C. None of the other climatic variables, either for summer or for the whole year, were associated with the size of the heat effect. In particular, higher summer mean temperature did not increase substantially the risk of mortality due to heat and whole year mean temperature was even less predictive of heat risk. This could suggest a partial adaptation to higher mean temperatures but not to the variation of summer temperatures. This almost agrees with previous studies conducted in US [1] that reported larger heat effects in cities with lower but more variable temperatures during warm months. Our finding of higher heat risk in cities with more varied summer temperatures is also in agreement with a recent multi-city study conducted in four US cohorts of older persons, which found that within cities, years with increased temperature variability were associated with increased the risk of mortality [23]. Also the CIRCE study has reported a similar finding, showing a higher heat risk in cities with larger mean, maximum and standard deviation of maximum apparent temperature, but not for higher mean temperatures [5].

Socio-economic factors have also been related with the variation in susceptibility to heat-effects in US [1,11,24,25]. In our framework, a study conducted in Barcelona at individual level concluded that the educational level was an important determinant of excess mortality in the 2003 heat wave [26]. In this sense our data was limited, since per capita income, as well as most of other highly correlated socio-economic variables, showed a more homogeneous distribution than those allowed in international studies. Although we found the same pattern it was not statistically significant. Similar findings were reported by the CIRCE study, where they did collected variables for health expenditure and unemployment, being both not significant [5].

The major strength of the study was the availability of long time series data sets from 50 provincial capital of Spain, providing enough power to identify and estimate heat effects across geographic, climatic conditions in Spain. Our 50-city dataset is matched only by those from the US [11] in being able to cover a wide variety of climates within one country. Besides the usual limitations that affect ecological time-series, as misclassification of exposure and absence of vulnerability factors at individual level, we did not incorporate variables that might modify or be part of the heat impact, like air pollution levels. Multi-city studies conducted in US showed that heat-effect was reduced by 15% after adjusting for ozone [1] and by 30% after control for ozone and particulate matter [24], even though associations of heat and mortality remained significant. We did not adjust for humidity, but there is little evidence that humidity is associated with mortality [27]. Finally, we did not attempt to distinguish heat effects for different causes of death, which is known that there are different degrees of sensitivity to high temperatures, neither to evaluate mortality displacement.

## Conclusions

Our study provides nation-wide quantitative estimates for the impact that that high summer temperatures have on daily mortality in Spain. Variability in high summer temperatures is associated with high risk of mortality due to heat. More attention needs to be paid to the variability of extreme temperatures as considering future climate change these are expected to increase.

## Competing interests

The authors declare they have no competing interests.

## Authors’ contributions

AT and BA conceived the study. AT analyzed the data in consultation with BA and AG. AT wrote the draft version and revisions of the manuscript according to the contribution of BA, AG and JD. All authors read and approved the final version of the manuscript.

## Supplementary Material

Additional file 1 City-specific temperature-mortality associations (natural cubic splines with 4 degrees of freedom; equal quantile knots) across 50 Spanish provincial capital cities (sorted by latitude, North to South). Click here for file

Additional file 2 City-specific percentage relative risks (%RR) of mortality, comparing the 99th to 90th percentile of maximum temperature and by 1°C increase (sorted by latitude, North to South), and overall estimate from a random effects meta-analysis. Click here for file

Additional file 3 Geographic, socio-demographic and climatic characteristics of continental capital cities in Spain (sorted by latitude, North to South). Click here for file

Additional file 4 Correlation matrix between geographic, socio-demographic and climatic characteristics of continental capital cities in Spain. Click here for file
