# Supplementary figures and images for: Effects of high summer temperatures on mortality in 50 Spanish cities
Source: Environ Health. 2014 Jun 9;13:48. doi: 10.1186/1476-069X-13-48 (PMC4078369; doi:10.1186/1476-069X-13-48)

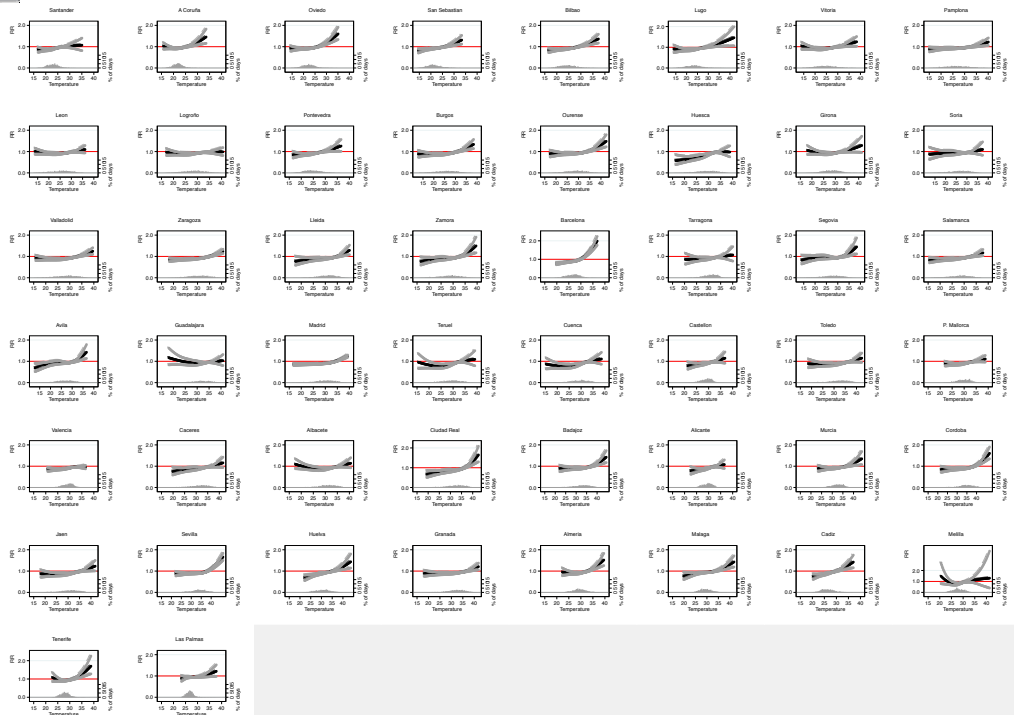

Supplement: Additional file 1 — City-specific temperature-mortality associations (natural cubic splines with 4 degrees of freedom; equal quantile knots) across 50 Spanish provincial capital cities (sorted by latitude, North to South). [file 1476-069X-13-48-S1.pdf]
