# Supplementary material for: Effects of high summer temperatures on mortality in 50 Spanish cities
Source: Environ Health. 2014 Jun 9;13:48. doi: 10.1186/1476-069X-13-48 (PMC4078369; doi:10.1186/1476-069X-13-48)
Supplement: Additional file 2 — City-specific percentage relative risks (%RR) of mortality, comparing the 99th to 90th percentile of maximum temperature and by 1°C increase (sorted by latitude, North to South), and overall estimate from a random effects meta-analysis. [file 1476-069X-13-48-S2.pdf]

City	Heat exposure as maximum temp. (ºC) (p90 , p99)	Dif.[a]	Risk of mortality comparing p99 vs. p90 %RR	(95% CI)	Risk of mortality by 1ºC increase %RR	(95% CI)
Santander	(24.5 , 28.1)	3.6	7.2	(1.7 , 13.0)	2.0	(0.5 , 3.5)
A Coruña	(23.0 , 26.6)	3.6	11.8	(7.1 , 16.6)	3.1	(1.9 , 4.3)
Oviedo	(23.9 , 28.1)	4.2	16.1	(11.4 , 20.9)	3.6	(2.6 , 4.6)
San Sebastian	(23.4 , 28.3)	4.9	15.8	(9.3 , 22.7)	3.0	(1.8 , 4.3)
Bilbao	(26.8 , 31.6)	4.8	16.4	(11.0 , 22.2)	3.2	(2.2 , 4.2)
Lugo	(25.7 , 31.9)	6.3	15.7	(8.0 , 23.9)	2.4	(1.2 , 3.5)
Vitoria	(27.0 , 32.9)	5.8	13.8	(6.0 , 22.2)	2.2	(1.0 , 3.5)
Pamplona	(29.4 , 35.2)	5.8	12.9	(6.2 , 20.0)	2.1	(1.0 , 3.2)
Leon	(27.9 , 32.6)	4.7	9.2	(0.9 , 18.2)	1.9	(0.2 , 3.6)
Logroño	(30.9 , 36.6)	5.7	3.7	(-5.4 , 13.6)	0.6	(-1.0 , 2.3)
Pontevedra	(26.8 , 32.0)	5.2	15.6	(6.1 , 25.9)	2.8	(1.1 , 4.5)
Burgos	(28.7 , 33.9)	5.2	19.1	(11.1 , 27.6)	3.8	(2.3 , 5.2)
Ourense	(31.6 , 37.1)	5.5	22.3	(13.5 , 31.8)	3.4	(2.1 , 4.8)
Huesca	(31.7 , 36.2)	4.5	4.5	(-9.9 , 21.2)	1.0	(-2.3 , 4.4)
Palencia[b]						
Girona	(30.3 , 34.6)	4.3	11.1	(3.0 , 19.9)	2.5	(0.7 , 4.3)
Soria	(29.3 , 34.0)	4.7	9.2	(-6.0 , 26.8)	1.9	(-1.3 , 5.2)
Valladolid	(31.1 , 36.0)	4.9	16.6	(9.9 , 23.8)	3.2	(2.0 , 4.4)
Zaragoza	(32.8 , 37.3)	4.5	16.9	(12.0 , 22.0)	3.5	(2.6 , 4.5)
Lleida	(33.0 , 37.1)	4.1	19.1	(8.8 , 30.4)	4.4	(2.1 , 6.7)
Zamora	(31.0 , 35.8)	4.8	26.8	(12.9 , 42.5)	5.1	(2.6 , 7.7)
Barcelona	(28.3 , 31.5)	3.3	27.3	(23.2 , 31.5)	7.7	(6.6 , 8.8)
Tarragona	(32.5 , 35.4)	2.9	5.0	(-3.3 , 14.1)	1.7	(-1.2 , 4.7)
Segovia	(30.0 , 34.5)	4.5	21.2	(8.3 , 35.5)	4.4	(1.8 , 7.0)
Salamanca	(30.5 , 35.4)	4.9	15.7	(7.0 , 25.1)	3.0	(1.4 , 4.7)
Avila	(28.9 , 33.3)	4.4	24.3	(10.6 , 39.6)	5.1	(2.3 , 7.9)
Guadalajara	(34.0 , 38.3)	4.4	6.3	(-8.3 , 23.4)	1.4	(-2.0 , 4.9)
Madrid	(32.2 , 36.5)	4.3	18.5	(15.5 , 21.5)	4.0	(3.4 , 4.6)
Teruel	(31.3 , 35.5)	4.2	11.1	(-4.5 , 29.3)	2.5	(-1.1 , 6.3)
Cuenca	(31.3 , 35.4)	4.1	11.7	(-1.1 , 26.2)	2.7	(-0.3 , 5.8)
Castellon	(30.5 , 32.9)	2.5	7.6	(1.1 , 14.6)	3.0	(0.4 , 5.7)
Toledo	(34.9 , 39.1)	4.1	12.2	(2.2 , 23.2)	2.8	(0.5 , 5.2)
P. Mallorca	(31.6 , 35.0)	3.4	8.9	(3.2 , 14.8)	2.5	(0.9 , 4.2)
Valencia	(30.7 , 33.6)	3.0	2.1	(-1.6 , 6.0)	0.7	(-0.5 , 2.0)
Caceres	(34.1 , 38.8)	4.7	14.2	(1.1 , 29.1)	2.8	(0.2 , 5.5)
Albacete	(33.1 , 37.0)	3.9	11.0	(1.0 , 22.0)	6.6	(4.0 , 9.2)
Ciudad Real	(34.7 , 38.7)	4.0	28.9	(17.0 , 42.0)	2.7	(0.3 , 5.2)
Badajoz	(35.3 , 39.9)	4.7	20.1	(11.1 , 29.9)	4.0	(2.3 , 5.8)
Alicante	(30.8 , 33.2)	2.4	6.8	(0.9 , 12.9)	2.8	(0.4 , 5.2)
Murcia	(34.1 , 37.2)	3.1	9.0	(4.7 , 13.6)	2.8	(1.5 , 4.2)
Cordoba	(36.9 , 41.6)	4.7	25.4	(17.6 , 33.7)	4.9	(3.5 , 6.3)
Jaen	(33.1 , 37.7)	4.6	14.5	(5.5 , 24.2)	3.0	(1.2 , 4.8)
Sevilla	(36.1 , 40.9)	4.7	26.4	(21.8 , 31.2)	5.1	(4.3 , 5.9)
Huelva	(32.7 , 37.7)	5.1	17.4	(9.5 , 25.8)	3.2	(1.8 , 4.6)
Granada	(34.3 , 38.5)	4.2	14.9	(7.7 , 22.7)	3.3	(1.8 , 5.0)
Almeria	(30.9 , 35.6)	4.7	26.2	(16.8 , 36.3)	5.1	(3.4 , 6.9)
Malaga	(30.9 , 34.9)	4.0	11.6	(7.3 , 16.0)	2.8	(1.8 , 3.8)
Cadiz	(27.9 , 32.6)	4.7	21.7	(12.5 , 31.8)	4.3	(2.6 , 6.1)
Ceuta[b]						
Melilla	(29.4 , 32.9)	3.5	19.5	(2.4 , 39.6)	5.2	(0.7 , 10.0)
Tenerife	(29.1 , 32.0)	2.9	13.8	(8.4 , 19.4)	4.6	(2.9 , 6.4)
Las Palmas	(27.8 , 30.8)	3.0	3.8	(-0.5 , 8.4)	1.3	(-0.2 , 2.7)
**Combined[c]**	**(30.5 , 34.8)**	**4.3**	**14.6**	**(12.5 , 16.8)**	**3.3**	**(2.8 , 3.6)**

[a] p99-p90 of max. temp. (ºC)

[b] Data on temperature not available

[c] All cities combined from a random effects meta-analysis
