# Supplementary material for: Effects of high summer temperatures on mortality in 50 Spanish cities
Source: Environ Health. 2014 Jun 9;13:48. doi: 10.1186/1476-069X-13-48 (PMC4078369; doi:10.1186/1476-069X-13-48)
Supplement: Additional file 3 — Geographic, socio-demographic and climatic characteristics of continental capital cities in Spain (sorted by latitude, North to South). [file 1476-069X-13-48-S3.pdf]

| | Geographic | | Socio-demographic | | | Climate (Whole year) | | (Summer) | |
|---|---|---|---|---|---|---|---|---|---|
| City | Latitude | Longitude | Pop.[b] | %>65[c] | PCI[d] | Mean temp. | Humidity | Mean temp. | Humidity |
| Santander | 43.3 | -3.5 | 184,165 | 18.7% | 10,231 € | 18.6 | 74.8 | 19.2 | 76.8 |
| A Coruña | 43.2 | -8.2 | 243,402 | 17.1% | 8,518 € | 17.9 | 74.7 | 18.7 | 76.2 |
| Oviedo | 43.2 | -5.5 | 200,453 | 18.7% | 9,616 € | 17.5 | 77.4 | 18.0 | 79.4 |
| San Sebastian | 43.2 | -1.6 | 179,208 | 18.9% | 12,788 € | 16.6 | 77.3 | 18.4 | 81.6 |
| Bilbao | 43.2 | -2.6 | 357,589 | 19.6% | 12,473 € | 19.5 | 71.6 | 19.6 | 72.4 |
| Lugo | 43.0 | -7.3 | 87,480 | 18.3% | 8,737 € | 17.5 | 77.9 | 17.2 | 73.9 |
| Vitoria | 42.5 | -2.4 | 217,154 | 14.1% | 12,785 € | 17.4 | 75.8 | 18.0 | 71.6 |
| Pamplona | 42.5 | -1.4 | 180,483 | 17.5% | 12,900 € | 18.4 | 66.4 | 19.9 | 59.1 |
| Leon | 42.4 | -5.3 | 139,809 | 19.4% | 9,152 € | 16.7 | 67.2 | 18.2 | 55.7 |
| Logroño | 42.3 | -2.3 | 127,093 | 16.1% | 11,943 € | 19.7 | 68.8 | 21.3 | 60.7 |
| Pontevedra | 42.3 | -8.4 | 74,139 | 14.8% | 9,057 € | 19.2 | 72.9 | 19.5 | 70.0 |
| Ourense | 42.2 | -7.5 | 109,120 | 17.7% | 9,009 € | 21.4 | 70.0 | 21.3 | 61.0 |
| Burgos | 42.2 | -3.4 | 162,802 | 16.3% | 11,288 € | 16.9 | 70.8 | 18.1 | 60.4 |
| Huesca | 42.1 | -0.2 | 45,627 | 18.7% | 11,380 € | 19.6 | 63.8 | 22.1 | 51.9 |
| Palencia[a] | 42.0 | -4.3 | 80,332 | 17.7% | 10,315 € | | | | |
| Girona | 41.6 | 2.5 | 72,682 | 15.6% | 13,076 € | 20.9 | 70.6 | 21.9 | 64.9 |
| Soria | 41.5 | -2.3 | 34,045 | 19.0% | 11,270 € | 17.3 | 64.5 | 18.7 | 54.4 |
| Valladolid | 41.4 | -4.4 | 319,998 | 15.2% | 10,561 € | 18.9 | 64.8 | 20.7 | 50.5 |
| Zaragoza | 41.4 | -0.5 | 603,367 | 17.7% | 11,076 € | 21.2 | 61.6 | 23.5 | 51.2 |
| Lleida | 41.4 | 0.4 | 112,207 | 16.9% | 13,058 € | 21.5 | 66.3 | 23.3 | 55.9 |
| Zamora | 41.3 | -5.5 | 64,906 | 18.1% | 8,729 € | 19.1 | 64.9 | 21.0 | 53.0 |
| Barcelona | 41.2 | 2.1 | 1,503,451 | 22.0% | 11,708 € | 20.3 | 70.2 | 22.7 | 68.4 |
| Tarragona | 41.2 | 1.2 | 113,016 | 15.3% | 11,923 € | 23.2 | 69.2 | 24.8 | 66.5 |
| Segovia | 40.6 | -4.1 | 54,175 | 18.1% | 10,799 € | 17.8 | 59.0 | 20.1 | 44.8 |
| Salamanca | 40.6 | -5.4 | 158,720 | 18.7% | 9,092 € | 18.8 | 65.1 | 19.8 | 53.0 |
| Avila | 40.4 | -4.4 | 47,682 | 16.4% | 9,148 € | 16.9 | 63.0 | 18.7 | 48.4 |
| Guadalajara | 40.4 | -3.1 | 69,959 | 14.3% | 8,636 € | 21.1 | 63.7 | 21.6 | 47.9 |
| Madrid | 40.2 | -3.4 | 2,879,052 | 19.4% | 12,781 € | 20.0 | 58.6 | 23.5 | 45.4 |
| Teruel | 40.2 | -1.1 | 30,047 | 19.3% | 10,660 € | 19.5 | 65.3 | 20.1 | 57.0 |
| Cuenca | 40.0 | -2.1 | 45,100 | 17.7% | 9,165 € | 19.3 | 61.2 | 21.4 | 48.3 |
| Castellon | 39.6 | -0.1 | 139,712 | 15.0% | 11,256 € | 22.5 | 66.5 | 24.3 | 65.5 |
| Toledo | 39.5 | -4.0 | 67,617 | 14.2% | 8,511 € | 22.2 | 59.7 | 24.6 | 44.2 |
| P. Mallorca | 39.3 | 2.4 | 326,993 | 14.7% | 12,503 € | 22.6 | 72.0 | 23.6 | 64.9 |
| Valencia | 39.3 | -0.2 | 739,412 | 17.3% | 10,082 € | 23.1 | 65.5 | 24.8 | 66.8 |
| Caceres | 39.3 | -6.2 | 78,614 | 13.5% | 7,539 € | 21.9 | 60.7 | 24.3 | 43.1 |
| Ciudad Real | 38.6 | -3.6 | 61,138 | 14.7% | 8,837 € | 21.9 | 60.0 | 24.6 | 44.3 |
| Albacete | 38.6 | -1.5 | 147,527 | 13.0% | 8,478 € | 20.8 | 60.6 | 22.8 | 48.9 |
| Badajoz | 38.5 | -6.6 | 136,613 | 12.3% | 7,545 € | 23.9 | 64.5 | 24.6 | 50.8 |
| Alicante | 38.2 | -0.3 | 272,432 | 16.2% | 9,697 € | 23.4 | 65.1 | 24.7 | 64.3 |
| Murcia | 37.6 | -1.1 | 353,504 | 13.8% | 8,736 € | 24.9 | 57.1 | 25.9 | 51.5 |
| Cordoba | 37.5 | -4.5 | 311,708 | 14.1% | 7,909 € | 25.1 | 61.3 | 26.1 | 47.1 |
| Jaen | 37.5 | -3.5 | 109,247 | 13.7% | 7,802 € | 21.4 | 59.0 | 25.0 | 47.2 |
| Sevilla | 37.2 | -5.6 | 701,927 | 14.7% | 7,997 € | 25.6 | 59.7 | 26.6 | 49.3 |
| Huelva | 37.2 | -6.6 | 140,583 | 12.7% | 8,508 € | 24.0 | 65.8 | 24.4 | 56.3 |
| Granada | 37.1 | -3.4 | 244,767 | 16.3% | 7,731 € | 22.4 | 57.7 | 23.9 | 45.0 |
| Almeria | 36.5 | -2.3 | 169,027 | 12.9% | 10,239 € | 23.4 | 65.0 | 25.3 | 62.8 |
| Malaga | 36.4 | -4.2 | 530,553 | 13.6% | 8,007 € | 23.3 | 65.5 | 24.6 | 60.6 |
| Cadiz | 36.3 | -6.2 | 142,449 | 14.2% | 7,750 € | 21.6 | 71.1 | 23.6 | 69.2 |

[a] Data on temperature and humidity not available

[b] Total population (Source: Demographic Information System, Municipal Register 1999, National Statistics Institute)

[c] Percentage of people older than 65 years (Source: Demographic Information System, Municipal Register 1999, National Statistics Institute)

[d] Per capita income (Source: Spanish Regional Accounts, Base 2000, National Statistics Institute)
