# Supplementary material for: Effects of high summer temperatures on mortality in 50 Spanish cities
Source: Environ Health. 2014 Jun 9;13:48. doi: 10.1186/1476-069X-13-48 (PMC4078369; doi:10.1186/1476-069X-13-48)
Supplement: Additional file 4 — Correlation matrix between geographic, socio-demographic and climatic characteristics of continental capital cities in Spain. [file 1476-069X-13-48-S4.pdf]

	Heat exp.	Geographic		Socio-demographic			Climate (Whole year)		(Summer)	
	p99-p90	Latitude	Longitude	Pop.[a]	%>65[b]	PCI[c]	Mean temp.	Humidity	Mean temp.	Humidity
**Heat expoure**										
p99-p90 of max. temp. (ºC)	1									
**Geographic**										
Latitude	0.46	1								
Longitude	-0.40	0.01	1							
**Socio-demographic**										
Population (x100,000 hab.)[a]	-0.12	-0.01	0.16	1						
% Pop. >65 years[b]	0.12	0.65	0.16	0.36	1					
Per capita income (x1,000 €)[c]	0.11	0.55	0.67	0.27	0.44	1				
**Climate**										
Whole year										
Mean temperature (ºC)	-0.40	-0.78	0.16	0.06	-0.61	-0.35	1			
Relative humidity (%)	0.71	0.63	-0.07	-0.13	0.29	0.35	-0.47	1		
Summer (Jun. To Sep.)										
Mean temperature (ºC)	-0.55	-0.81	0.24	0.16	-0.57	-0.30	0.95	-0.62	1	
Relative humidity (%)	0.61	0.48	0.06	-0.04	0.28	0.36	-0.30	0.92	-0.44	1

[a.] Total population (Source: Demographic Information System, Municipal Register 1999, National Statistics Institute)

[b.] Percentage of people older than 65 years (Source: Demographic Information System, Municipal Register 1999, National Statistics Institute)

[c.] Per capita income (Source: Spanish Regional Accounts, Base 2000, National Statistics Institute)
